# Feasibility of salivary cortisol collection in patients and companions attending dementia diagnostic meetings in memory clinics

**DOI:** 10.1186/s13104-021-05446-6

**Published:** 2021-01-21

**Authors:** H. Pavlickova, A. E. Russell, S. Lightman, R. McCabe

**Affiliations:** 1 Mental Health Research Group, University of Exeter Medical School, University of Exeter, Exeter, UK; 2 Henry Wellcome Laboratories for Integrative Neuroscience and Endocrinology, Dorothy Hodgkin Building, University of Bristol, Bristol, BS1 3NY UK; 3 School of Health Sciences, City, University of London, 1 Myddelton Street, London, EC1R 1UW UK

**Keywords:** Cortisol, Dementia, Diagnostic meeting, Memory clinic

## Abstract

**Objectives:**

Receiving a diagnosis of dementia is life-changing for the individual and their companion. The aim of the study was to explore the feasibility of collecting salivary cortisol from patients who are informed if they have dementia and their companions. Patients and companions collected nine saliva samples in three batches: 1–2 weeks before, immediately before, and immediately after the diagnostic meeting. Each batch consisted of three samples taken in the evening, after awaking and 30 mins post-waking.

**Results:**

22.7% (N = 10) of 44 invited patients and nine companions agreed, with 18.2% patients (N = 8) and 15.9% companions (N = 7) providing samples. Participants found that saliva collection was demanding and disrupted routines. On a purely descriptive level, some indications of an increased cortisol stress response in patients diagnosed with dementia were found in this very small sample. Researchers should expect low recruitment rates in this elderly population. Simpler collection procedures, e.g. pre-labelled packages with date/time, possible omission of morning samples and objective rather than self-report assessment of waking and saliva collection times—using actigraphy wrist-watches bleeps to prompt people at the timepoints and electronic track caps—might improve adherence and improve the accuracy of timepoints when swabs were actually collected.

## Introduction

Receiving a diagnosis of dementia is life changing for patients and their companions. It involves facing progressive cognitive and physical symptoms, concerns about legal issues (e.g. power of attorney) and living arrangements, in particular considering moving into a care home away from family. Dementia is highly stigmatised and is the most feared illness among over 55 s [1].

One way of investigating the impact of a dementia diagnosis is measuring the hormone cortisol implicated in the bodily response to stress [2, 3]. Cortisol is most commonly assessed from saliva due to its non-invasiveness and laboratory independence. This requires participants’ compliance with collection protocols including repeated sampling, and refraining from smoking, eating, or brushing teeth to prevent saliva contamination.

Previous literature has examined the role of cortisol in cognitive decline and dementia [4–6] and as a marker of stress in carers of individuals with dementia [7]. However, to our knowledge, no study has measured cortisol when receiving a diagnosis of dementia or other life-limiting conditions.

While understanding the impact of the diagnosis on a physiological level might be informative, adhering to a strict saliva collection protocol might be challenging in this population who present with memory impairment and other cognitive difficulties. Some patients live alone and others rely on considerable support from spouses or other family members/friends who may also be elderly and have their own healthcare needs. Asking patients and companions to collect saliva samples in this situation might present additional challenges.

Hence, the aim of the study was to examine the feasibility [8] of collecting saliva samples before and after attending a diagnostic feedback meeting in the memory clinic where the patient is informed if they have dementia or not. Cortisol levels in both patients and companions were also assessed.

## Main text

### Methods

#### Setting

The study was part of a NIHR-funded project ‘*Shared decision making in mild to moderate dementia (ShareD)*’ examining patient involvement in shared decision making in diagnostic meetings in memory clinics. The meeting was to inform patients if they had dementia or not. The study was conducted across nine memory clinics in two U.K. locations—London and Devon—with ethical approval from Camden and Islington Research Ethics Committee (13/LO/1309).

#### Recruitment

A subsample of participants, i.e., patients and their companions (relatives/friends), with a scheduled diagnostic feedback meeting were invited to collect saliva samples. Having a companion was not necessary for participation. The only exclusion criteria were: needing an interpreter or if companions were paid. Recruitment took place from 01/03/2017 to 28/04/2017.

Patients were sent a letter by the researcher and contacted by phone several weeks before their memory clinic meeting. The researcher visited interested patients and companions at home to provide detailed information, obtain informed consent and provide saliva collection instructions.

#### Saliva collection

Patients and companions each received nine pre-labelled tubes and cotton swabs to collect saliva samples. Participants were asked to place a cotton swab under their tongue for 2 min. Saliva samples were collected in three batches taken in the evening, the following morning on waking, and 30 min thereafter. The first batch of samples was collected 1–2 weeks before the diagnostic feedback meeting (i.e. baseline); the second batch was collected on the night before and the morning of the diagnostic meeting; and the third batch on the night of and morning after the diagnostic meeting.

The researcher explained the procedures to participants and provided detailed written instructions. Patients/companions were asked if they wanted reminder calls: one pair wanted reminder calls. The tubes had barcode labels with the timepoint (baseline, night before clinic, night after clinic) and time of day (evening, wakeup, 30 min after wakening) when saliva was to be collected. All nine samples were collected together by the researcher and participants reimbursed with £20. Adherence to the instructions were assessed in based on researcher interviews with the patients/companions when collecting the samples.

Participants were instructed to refrain from smoking, eating, or brushing their teeth for at least 30 min before, and consuming alcohol for 12 h before cortisol collection. They were asked to store samples in their home freezer.

Participants’ reports regarding the feasibility of self-collection of saliva samples around diagnostic meetings at memory clinics were analysed descriptively.

Saliva samples were stored in accordance with Human Tissue Act regulations in a − 80 °C freezer at the University of Exeter Medical School until shipped for analyses. Samples were frozen at − 80 °C and analysed in duplicate at ARU Biomarker Laboratory, Cambridge, UK. Cortisol levels were determined using a commercially available competitive ELISA (Salimetrics LLC, Carlsbad, CA, USA). Samples were thawed, vortexed and centrifuged at 1500×*g* for 15 min and clear samples pipetted into appropriate wells. Samples were thawed and reconstituted with 0.125 mL of Salimetrics cortisol assay diluents. The inter-assay variability calculated from the average CVs of high and low controls was 5.7% (n = 10). The intra-assay variability was calculated from the average CVs for the assay as 5.68% (n = 133).

#### Cortisol variables

Three composite indices were calculated to describe the diurnal cortisol profile: Diurnal cortisol slope (DCS), Area under the curve with respect to ground (AUC_G_), and Cortisol Awaking Response (CAR).

DCS was calculated by subtracting cortisol bedtime values from values immediately after waking and dividing by the number of hours between the two samples [9]. The following formula was used to calculate AUC_G_: number of hours between bedtime and wakeup samples × (cortisol concentration at bedtime + cortisol concentration at wakeup)/2 [10]. As participants did not record the exact collection time of each sample, we estimated 8 h as a distance between samples for both DCS and AUC_G_ calculations.

CAR was calculated as the ratio of the post-awakening sample to wakeup sample [11].

#### Data analyses

Given the feasibility nature of the study and a small data set, cortisol levels were analysed descriptively. Normality tests were not performed. Statistical analyses were performed in SPSS 24 and Stata 12.

### Results

44 patients and their companions were invited to take part. Ten out of 45 patients agreed: the patient consent rate was 22.7%. Eight companions consented. One patient was living alone so had no companion to be approached**.** Eight participants (18.2%) and seven companions (15.9%) collected saliva samples. Participant flow is available from authors on request.

#### Participant socio-demographic and clinical characteristics

Socio-demographic and clinical characteristics of participants and their companions who collected saliva samples are presented in Table 1.

**Table 1:** Participant socio-demographic and clinical information

	Patient (N = 8) Mean (SD or %)	Companion (N = 7) Mean (SD or %)
Age	68.5 (13.2)	61.8 (13.33)
Sex		
Female	4 (50.0%)	4 (57.1%)
Male	4 (50.0%)	3 (42.9%)
Marital status		
Married/partnership	5 (62.5%)	6 (85.7%)
Single	0 (0%)	0 (0%)
Divorced	1 (12.5%)	0 (0%)
Widowed	2 (25.0%)	1 (14.3%)
Ethnicity		
White British	7 (87.5%)	0 (0%)
White and Black Caribbean	1 (12.5%)	7 (100%)
Companion relationship		
Spouse	5 (62.5%)	
Daughter/son	1 (12.5%)	
Friend	2 (25.0%)	
Diagnosis		
Alzheimer’s disease	2 (25.0%)	
Mild cognitive impairment	3 (37.5%)	
Anxiety or stress	1 (12.5%)	
No diagnosis—further tests	2 (25.0%)	

Two of the eight participants completing cortisol collection received a diagnosis of dementia at the memory clinic. Six participants did not have dementia (see Table 1 for more detail), including two participants who were invited for further tests to determine the diagnosis.

#### Feasibility of self-administered saliva collection in memory clinic patients and their companions

##### Reasons for not taking part in cortisol collection

77.2% (N = 34) patients declined to participate. They gave reasons including: saliva collection was too burdensome; feeling concerned about the memory clinic appointment; or being too unwell. While we could not collect systematic data on the characteristics of participants who declined as we did not have ethical approval, anecdotally they found the salivary collection procedures confusing.

##### Participant feedback on process

Participants were elderly and most of them were supported in saliva collection by live-in companions (i.e. spouse/child). Although participants were offered telephone reminders from the research team, all except one pair declined.

All participants provided feedback. They reported:

- Collection instructions were confusing, such as matching individual swabs with prelabelled tubes;
- Difficulties abstaining from alcohol. Of the eight participants who collected saliva samples at least two reported drinking alcohol on the days of the swabs;
- Swabs were extremely dry in the mouth;
- Disruptions to established morning routines—waiting half an hour after waking before eating/drinking/teeth brushing—and evening routines—abstaining from alcohol;
- Participants with an early appointment at the memory clinic had to wake up extremely early for the two morning samples;
- One couple reported that they needed a mirror to see where the swab was in their mouth to accurately spit it into the tube.

#### Cortisol

##### Cortisol data cleaning

Cortisol samples were excluded if (i) evening concentrations of cortisol were higher than those at awaking and/or post-awaking as this is not physiologically feasible, and most likely caused by mislabelling or mixing up tubes (six samples); (ii) samples were of the same low value (seven samples). This is likely to be caused by collecting samples at the same time in the evening. In addition, two samples were treated as missing due to the lack of sufficient saliva for analyses. Values of 1.0 nmol/L were assigned to three cortisol samples with levels below the minimum detectable level for the assay (1.0 nmol/L). Hence, not all samples obtained could be analysed: 18 samples were not analysed either due to mixing up of times or lack of sufficient saliva for analysis. After data cleaning, there were 133 observations.

##### Cortisol levels in patients with and without dementia and companions

**Fig. 1.**
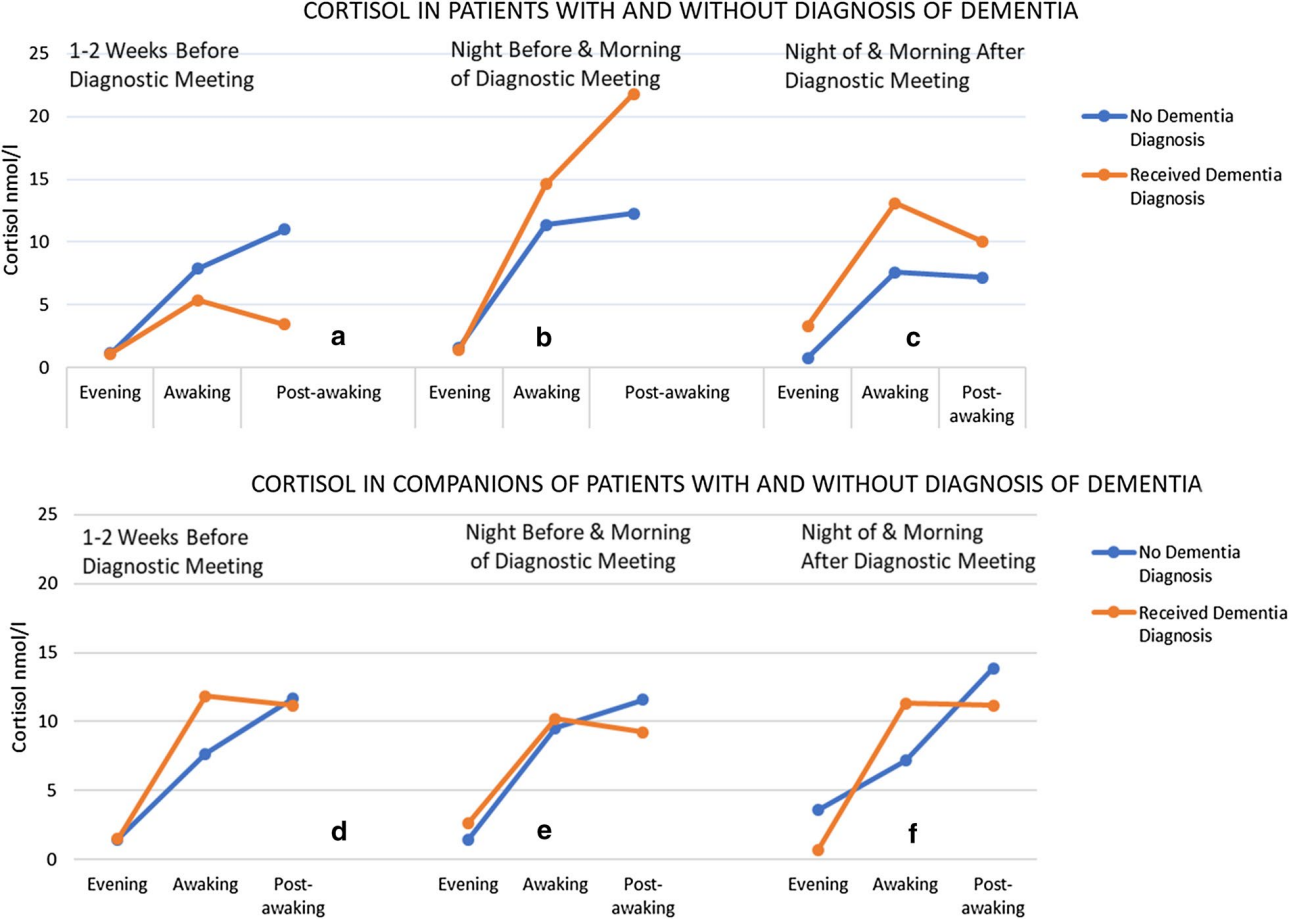
Cortisol in patients with and without a diagnosis of dementia

On a descriptive level, baseline cortisol levels were lower in participants diagnosed with dementia than those without dementia. Cortisol levels around the diagnostic meeting were higher in patients who received the diagnosis of dementia (M = 8.25, SD = 2.38), compared to those who did not (M = 5.94, SD = 4.38)—see panels b and c in Fig. 1. In companions, there were less pronounced differences between the groups (in companions of patients with dementia M = 7.76, SD = 1.65 compared to companions of patients without dementia M = 7.56, SD = 3.73)—see panels e and f in Fig. 1.

The composite values (DCS, AUCG and CAR) support the above findings (Table 2). Higher DCS and AUC_G_ values were found the night before and the night after the diagnostic meeting in patients who received a diagnosis of dementia, while CAR was decreased after the diagnostic meeting. Due to the heterogeneity of non-dementia diagnoses (mild cognitive impairment, anxiety and no diagnosis) limited sample size, these findings are presented in a purely descriptive manner.

**Table 2:** Diurnal cortisol slope (DCS), area under curve with respect to ground (AUC_G_) and Cortisol Awaking Response (CAR) 1–2 weeks before the diagnostic meeting, night before and night after diagnostic meeting in patients with and without a diagnosis of dementia and companions

	Baseline (Nmol/L) Mean (SD)			Before diagnostic meeting (Nmol/L) Mean (SD)			After diagnostic meeting (Nmol/L) Mean (SD)		
	DCS	AUC_G_	CAR	DCS	AUC_G_	CAR	DCS	AUC_G_	CAR
Patients diagnosed with dementia	0.53 (0.61)	25.94 (22.60)	1.12 (0.99)	1.66 (0.29)	64.02 (15.58)	1.39 (0.90)	1.22 (0.76)	65.66 (8.57)	0.75 (0.08)
Patients without diagnosis	0.94 (0.47)	40.55 (15.06)	1.23 (0.56)	1.00 (1.10)	44.99 (35.38)	1.20 (0.34)	0.85 (0.61)	33.38 (21.28)	0.81 (0.10)
Companions of patients diagnosed with dementia	1.29 (0.27)	53.50 (13.27)	0.95 (0.04)	0.95 (0.46)	51.32 (0.79)	0.95 (0.43)	1.33 (0.61)	48.02 (21.07)	1.05 (0.32)
Companions of patients without diagnosis	0.79 (0.27)	36.06 (12.57)	1.38 (0.32)	1.01 (0.64)	43.91 (19.35)	1.24 (0.32)	0.77 (0.42)	34.48 (17.20)	1.67 (0.99)

### Discussion

There are significant challenges to collecting salivary cortisol at home in this population. Over 75% of patients approached to participate in the study on cortisol in relation to diagnostic visits declined. Over 90% of consenting participants collected samples assisted by a family member/friend. However, adhering to the collection protocol—particularly the morning samples—was challenging as it impacted on participants’ established routines and a number of samples could not be analysed.

Future studies with older adults referred to memory clinics investigating diagnosis related stress should be aware that there is likely to be a low recruitment rate and problems with the integrity of the samples obtained. Given the demands on protocol adherence in cortisol studies and the circumstances of this population, minimising the burden on participants is important. This is aided by simple instructions, and pre-labelled packages, clearly indicating the date/time swabs are to be taken. Some studies might omit the morning samples: while they require timely collection [12], they were reported as most intrusive and difficult to complete. Another option might be to provide actigraphy wrist-watches pre-programmed with bleeps to prompt collection of samples. Rather than relying purely on self-report, more reliable data on waking and saliva collection times could be obtained using a combination of actigraphy wrist-watches and electronic track caps. However, this population may need assistance to collect reliable samples and to maximise the integrity of the samples.

## Limitations

Precise times of saliva sample collection were not collected. Further limitations include lack of documentation of awakening time and possible delays in sampling which may not have captured the CAR, along with estimating 8 h between bedtime and wakeup samples. Moreover, subjective measures of participant stress were not administered due to additional participant burden. As participants who needed an interpreter could not participate, the findings are limited in their generalizability to non-English speaking participants. Participants likely to receive a diagnosis of dementia are probably less likely to participate. Two participants were invited for further tests: for the present purposes, they were treated as participants without dementia, which might have introduced some noise. Ideally, in a larger sample these participants would be treated as a distinct category.

## Data Availability

The datasets generated and/or analysed during the current study are available from the corresponding author on reasonable request.

## References

[1] Kinzer A, Suhr JA (2016). Dementia worry and its relationship to dementia exposure, psychological factors, and subjective memory concerns. Appl Neuropsychol Adult.

[2] Dickerson SS, Kemeny ME (2004). Acute stressors and cortisol responses: a theoretical integration and synthesis of laboratory research. Psychol Bull.

[3] Kirschbaum C, Hellhammer DH (1994). Salivary cortisol in psychoneuroendocrine research: recent developments and applications. Psychoneuroendocrinology.

[4] Li G, Cherrier MM, Tsuang DW, Petrie EC, Colasurdo EA, Craft S (2006). Salivary cortisol and memory function in human aging. Neurobiol Aging.

[5] Hatfield CF, Herbert J, van Someren EJW, Hodges JR, Hastings MH (2004). Disrupted daily activity/rest cycles in relation to daily cortisol rhythms of home-dwelling patients with early Alzheimer’s dementia. Brain.

[6] Wahbeh H, Kishiyama SS, Zajdel D, Oken BS. Salivary cortisol awakening response in mild alzheimer disease, caregivers, and noncaregivers. Alzheimer Dis Assoc Disord. 2008;22:181–3. http://content.wkhealth.com/linkback/openurl?sid=WKPTLP:landingpage&an=00002093-200804000-00015. Accessed 5 Dec 2017. 10.1097/WAD.0b013e31815a9dff 18525292

[7] De Vugt ME, Nicolson NA, Aalten P, Lousberg R, Jolle J, Verhey FR (2005). Behavioral problems in dementia patients and salivary cortisol patterns in caregivers. J Neuropsychiatry Clin Neurosci.

[8] Orsmond GI, Cohn ES (2015). The distinctive features of a feasibility study: Objectives and guiding questions. OTJR Occup Particip Health.

[9] Adam EK, Kumari M (2009). Assessing salivary cortisol in large-scale, epidemiological research. Psychoneuroendocrinology.

[10] Clow A, Hucklebridge F, Stalder T, Evans P, Thorn L (2010). The cortisol awakening response: more than a measure of HPA axis function. Neurosci Biobehav Rev.

[11] Cohen S, Schwartz JE, Epel E, Kirschbaum C, Sidney S, Seeman T. Socioeconomic status, race, and diurnal cortisol decline in the Coronary Artery Risk Development in Young Adults (CARDIA) Study. Psychosom Med. 2006;68:41–50. http://kungfu.psy.cmu.edu/~scohen/Cohenetal2006PsychosomMed.pdf. Accessed 27 Nov 2017. 10.1097/01.psy.0000195967.51768.ea 16449410

[12] Clow A, Thorn L, Evans P, Hucklebridge F (2004). The awakening cortisol response: methodological issues and significance. Stress.

